# Adjunctive antimicrobial chemotherapy based on hydrogen peroxide photolysis for non-surgical treatment of moderate to severe periodontitis: a randomized controlled trial

**DOI:** 10.1038/s41598-017-12514-0

**Published:** 2017-09-25

**Authors:** Taro Kanno, Keisuke Nakamura, Kirika Ishiyama, Yasutomo Yamada, Midori Shirato, Yoshimi Niwano, Chie Kayaba, Koji Ikeda, Airi Takagi, Takuhiro Yamaguchi, Keiichi Sasaki

**Affiliations:** 1 Division of Molecular and Regenerative Prosthodontics, Tohoku University Graduate School of Dentistry, 4-1 Seiryo, Aoba-ku, Sendai, 980-8575 Japan; 2 Laboratory for Redox Regulation, Tohoku University Graduate School of Dentistry, 4-1 Seiryo, Aoba-ku, Sendai, 980-8575 Japan; 3 Sweden Dental Sendai (private practice), 1-6-2 Tsutsujigaoka, Miyagino-ku, Sendai, 983-0852 Japan; 4 Clinical Research, Innovation and Education Center, Tohoku University Hospital, 1-1 Seiryo, Aoba-ku, Sendai, 980-8574 Japan; 5 Clinical Research Data Center, Tohoku University Hospital, 1-1 Seiryo, Aoba-ku, Sendai, 980-8574 Japan; 6 Division of Advanced Prosthetic Dentistry, Tohoku University Graduate School of Dentistry, 4-1 Seiryo, Aoba-ku, Sendai, 980-8575 Japan

## Abstract

Treatment of severe periodontitis with non-surgical therapy remains challenging in dentistry. The present study aimed to evaluate the clinical efficacy of hydrogen peroxide (H_2_O_2_) photolysis-based antimicrobial chemotherapy adjunctively performed with root debridement (RD) for moderate to severe periodontitis. A randomized controlled trial was conducted that included 53 patients with 142 test teeth. The test teeth were randomly assigned to one of three treatment groups: Group 1, RD + H_2_O_2_ photolysis; Group 2, RD followed by administration of a local drug delivery system (minocycline chloride gel); or Group 3, RD alone. Clinical and microbiological examination were performed for up to 12 weeks following treatment. Probing pocket depth (PPD) and bleeding on probing (BoP) were improved after each treatment session. At 12 weeks, Group 1 had achieved significantly lower PPDs than the other groups, though there were no significant differences in BoP between Group 1 and the other groups. Counts of *Porphyromonas gingivalis*, a known periodontal pathogen, in Group 1 were significantly lower than those in Group 3, and were comparable to those in Group 2. Therefore, it is suggested that H_2_O_2_ photolysis treatment can be used as a novel adjunctive antimicrobial chemotherapy for non-surgical periodontal treatment.

## Introduction

Periodontitis is an inflammatory disease caused by pathogenic microorganisms in dental plaque (microbial biofilm), resulting in periodontal pocket formation, and loss of periodontal attachment and alveolar bone around the tooth^1–3^. Thus, periodontal therapy by mainly mechanical instrumentation, such as debridement, scaling and root planing (SRP), is performed to reduce the microbial load and/or disrupt microbial biofilm. Kieser proposed that debridement (defined as instrumentation for disruption and removal of microbial biofilms) should be assessed before proceeding to more aggressive instrumentations, such as scaling (instrumentation for removal of mineralized deposits) and root planing (instrumentation for removal of contaminated cementum and dentin)^4^. In this context, mechanical debridement using an ultrasonic scaler is widely performed, which removes less tooth structure than hand instruments^5,6^. A number of clinical studies have demonstrated that non-surgical periodontal therapy, including less-invasive debridement, is an effective treatment modality^7–9^ when accompanied by proper oral hygiene control. However, deep periodontal pockets tend to respond poorly to non-surgical therapy in comparison with shallow pockets^10^.

Mechanical debridement can be supplemented with antimicrobial chemotherapy, such as systemic antibiotics and topical application of antimicrobials^11,12^. However, improper use of systemic antibiotics could be accompanied by side effects, such as gastrointestinal problems, and the emergence of antibiotic resistant bacteria. As such, prescription of systemic antibiotics should be cautiously considered. To reduce the risk of systemic side effects, antibiotics incorporated into sustained-release vehicles (e.g., gels) at lower doses than in systemic administration have been developed and clinically applied; this method is known as a local drug delivery system (LDDS)^12–14^. Although multiple meta-analyses have shown that the use of LDDSs in conjunction with mechanical debridement improves periodontal conditions more than debridement alone^12,14^, the possibility of inducing bacterial resistance to the antibiotics cannot be ruled out. In addition, an LDDS usually requires multiple applications at specific intervals to ensure adequate exposure time of the subgingival bacteria to the drug^15,16^, which may be inconvenient or result in poor patient compliance.

To overcome the shortcomings of LDDSs, a novel antimicrobial technique, in which hydroxyl radicals generated by hydrogen peroxide (H_2_O_2_) photolysis act as the active ingredient, has been developed in our laboratory^17^. Since hydroxyl radicals are powerful oxidizing agents, they cause lethal oxidative damage to microorganisms. Hydroxyl radicals cannot be formulated into a ready-made disinfectant due to their very short life in a liquid medium (approximately 10^−9^ s)^18,19^. Instead, they can be generated by irradiating 3% H_2_O_2_ inside the periodontal pocket with light at a wavelength of 405 nm (i.e., photolysis)^17,20^. Since the light and H_2_O_2_ can penetrate the microbial biofilm, this antimicrobial technique is effective against biofilm-forming bacteria^21^. The bactericidal effect is strong, killing biofilm-forming bacteria with a > 4-log reduction of viable counts within one minute, and as such, a single treatment session may be sufficient for periodontal therapy. Furthermore, an *in vitro* study has suggested that repeated treatment using H_2_O_2_ photolysis does not induce bacterial resistance^22^. This is likely due to non-selective damage to bacterial cells caused by the hydroxyl radicals^23,24^. Hence, this technique is expected to be an effective alternative to LDDS for periodontal therapy.

Concerning the safety of H_2_O_2_ photolysis treatment, the Food and Drug Administration of the United States considers the application of 3% H_2_O_2_ to oral mucosa “acceptable”^25^, and light at 405 nm is within the range of visible light. In addition, it has been demonstrated that exposure of the oral mucosa to hydroxyl radicals generated by 3% H_2_O_2_ photolysis for a short time does not cause abnormal histological changes^26,27^. Based on these pre-clinical studies, we have developed a therapeutic device, named RP-14, that can perform ultrasonic root debridement (RD) concomitantly with H_2_O_2_ photolysis-based antimicrobial treatment. In this context, we hypothesized that 1) RD + H_2_O_2_ photolysis treatment would not result in inferior improvement of the periodontal condition in comparison to RD + LDDS treatment for moderate to severe periodontitis, and 2) RD + H_2_O_2_ photolysis treatment would improve the periodontal condition more than RD alone. Therefore, the aim of the present study was to evaluate the clinical efficacy and safety of RD + H_2_O_2_ photolysis treatment by testing the formulated hypotheses.

## Results

This randomized controlled trial was conducted from July 2015 to April 2016. Following the screening examination, 53 patients with 142 test sites were enrolled. The patients’ demographic data and the clinical characteristics of the test sites are presented in Table 1. The mean age of the patients was 55.5 years (standard deviation: 8.2). Forty-three patients were treated at the university hospital, while 10 were treated at the private clinic. There were no significant differences in probing pocket depth (PPD) and bleeding on probing (BoP) at the test sites between the groups at baseline (Table 2). The initial PPDs for Groups 1 (RD + H_2_O_2_ photolysis treatment), 2 (RD + LDDS treatment), and 3 (RD alone) were 6.76, 6.88, and 6.93 mm, respectively, while BoP was 100% for all groups. Fifty-two of 53 patients completed the 12-week study period. One patient, having three test sites, was withdrawn from follow-up because antibiotic administration became necessary due to development of acute periodontitis at a non-test site. At site level, treatment of a maxillary second molar site in Group 1 was discontinued because a perforation of the sinus membrane was observed during treatment; this test site was thus excluded from analysis owing to lack of data after treatment. Therefore, the intention-to-treat analysis was performed using data obtained from 141 test sites (Group 1, *n* = 48 sites; Group 2, *n* = 46 sites; and Group 3, *n* = 47 sites).

**Table 1:** Patient demographic data and clinical characteristics of the test sites.

	All groups	Group 1	Group 2	Group 3
**Number of patients**	53	49	46	47
Age, mean (years)	55.5	55.0	54.7	55.3
Gender (n)				
Male	18	17	16	18
Female	35	32	30	29
Facility (n)				
University clinic	43	41	41	40
Private clinic	10	8	6	6
**Number of test teeth**	142	49	46	47
Tooth types (n)				
Molar	27	10	9	8
Non-molar	115	39	37	39
Vertical bone loss (n)				
(+)	54	19	17	18
(−)	88	30	29	29
Location (n)				
Maxilla	74	26	23	25
Mandible	68	23	23	22

**Table 2:** Clinical assessment results.

		Group 1	Group 2	Group 3	P-value	
		Mean (95% CI)	Mean (95% CI)	Mean (95% CI)	Group 1 vs. Group 2	Group 1 vs. Group 3
PPD (mm)	BL	6.76	6.88	6.93	0.333	0.198
		(6.56–6.97)	(6.68–7.09)	(6.73–7.13)		
	4 weeks	4.95	5.67	5.46	<0.001	0.006
		(4.69–5.22)	(5.37–5.97)	(5.17–5.74)		
	8 weeks	4.74	5.14	5.11	0.030	0.055
		(4.45–5.02)	(4.85–5.43)	(4.79–5.42)		
	12 weeks	4.63	5.17	5.03	<0.001	0.013
		(4.39–4.87)	(4.84–5.50)	(4.78–5.29)		
BoP (%)	BL	100.0	100.0	100.0	—	—
		(−)	(−)	(−)		
	4 weeks	69.6	48.8	59.9	0.037	0.295
		(56.2–82.9)	(33.9–63.6)	(45.5–74.3)		
	8 weeks	52.2	37.3	37.7	0.130	0.141
		(37.7–66.7)	(23.0–51.7)	(23.4–51.9)		
	12 weeks	37.0	38.2	44.4	0.909	0.378
		(22.9–51.0)	(23.7–52.6)	(29.8–58.9)		

Group 1: root debridement + H_2_O_2_ photolysis. Group 2: root debridement + local drug delivery system. Group 3: root debridement alone. Abbreviations: BL, baseline; BoP, bleeding on probing; CI: confidence interval; PPD, probing pocket depth.

Clinical examination showed that the plaque index (PlI) at the test sites in all treatment groups was maintained at ≤ 1 throughout the study period. During the treatment phase, RD was performed for 341 s (95% confidence interval [CI]: 319–363) in Group 1, 341 s (95% CI: 296–345) in Group 2, and 318 s (95% CI: 293–343) in Group 3. There was no significant difference in the treatment time between groups. Reduction of PPD and BoP occurred after treatment (Table 2). At the 12-week examination, the mean PPDs in Groups 1, 2, and 3 were 4.63 mm, 5.17 mm, and 5.03 mm, respectively. The difference in PPD between Groups 1 and 2 was 0.54 mm, and the lowest value of the 95% CI (0.23 mm) was higher than the non-inferiority margin of -0.3 mm. Furthermore, the PPDs in Group 1 were significantly smaller than those recorded in Groups 2 (P < 0.001) and 3 (P = 0.013). Throughout the study period, the PPDs in Group 1 were significantly smaller than those in Groups 2 and 3, except for the difference between Groups 1 and 3 at the 8-week examination (P = 0.055). Regarding BoP, the percentage of positive sites in Groups 1, 2, and 3 at the 12-week examination was 37.0, 38.2, and 44.4%, respectively. There was no significant difference in BoP at 12 weeks between the groups (P > 0.05). A significant difference in BoP was observed only at the 4-week examination between Groups 1 and 2 (P = 0.037).

**Figure 1:**
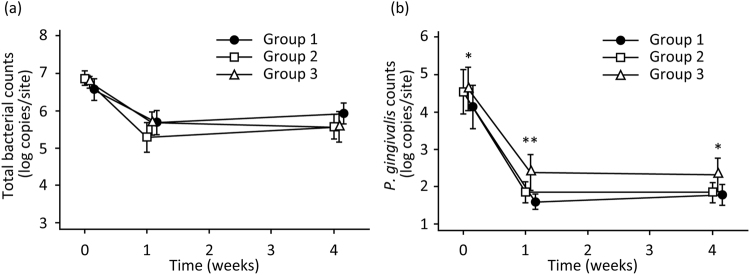
Changes in the total bacterial (**a**) and *Porphyromonas gingivalis* (**b**) counts after each treatment. Microbiological analysis was conducted with the invader-PCR technique. The values and error bars represent the means and 95% confidence intervals, respectively. Significant differences detected between Groups 1 and 3 are denoted by *P < 0.05 and **P < 0.01. Group 1, root debridement + H_2_O_2_ photolysis; Group 2, root debridement + local drug delivery system; and Group 3, root debridement alone.

Microbiological analysis showed that total bacterial counts decreased after treatment (Fig. 1a). Although Group 2 exhibited a trend of lower values than Group 1, there was no significant difference between the groups at each time point. The *P. gingivalis* counts also decreased after treatment. Group 1 had significantly lower bacterial counts than Group 3 at baseline (P = 0.029), and at the 1-week (P = 0.002) and 4-week (P = 0.015) examinations, whereas there was no significant difference between Groups 1 and 2 at any time point (Fig. 1b).

A laboratory test demonstrated that ultrasonic debridement against an acrylic resin plate with the hollow-type scaler tip used in Group 1 and the solid-type scaler tip used in Groups 2 and 3 decreased the weight of the plate. Debridement using a hollow-type scaler tip decreased the weight by 5.8 mg (95% CI: 4.5–7.1), whereas the solid-type scaler tip decreased it by 11.4 mg (95% CI: 9.7–13.0); the difference was statistically significant (P < 0.0001).

In total, 28 adverse events in 24 patients were reported and categorized according to the National Cancer Institute Common Terminology Criteria for Adverse Events (NCI CTCAE v4.0)^28^. Twenty-four events were categorized as Grade 1 (mild; asymptomatic or mild symptoms; clinical or diagnostic observations only; intervention not indicated), while four events were classified as Grade 2 (moderate; minimal, local, or non-invasive intervention indicated; limiting age-appropriate instrumental activities of daily living). No event categorized as Grade 3 or higher occurred. Of the 28 adverse events, 26 were not directly related to the treatment of test teeth. These included transient postoperative pain (*n* = 9), stomatitis (*n* = 2), hypersensitivity (*n* = 2), acute periodontitis (*n* = 2) etc., which developed at non-test sites. Two adverse events were possibly correlated with the test treatment. One was perforation of the sinus membrane observed during the treatment phase in Group 1, and the other was stomatitis that developed at the edge of the tongue close to a test site in Group 1.

## Discussion

The present randomized controlled trial evaluated the effect of a new non-surgical therapy for moderate to severe periodontitis, consisting of RD with concomitant antimicrobial chemotherapy based on H_2_O_2_ photolysis using the newly developed RP-14 device. The results demonstrated that RD + H_2_O_2_ photolysis treatment (Group 1) was not significantly inferior to RD + LDDS treatment (Group 2) regarding the primary outcome (PPD at the 12-week examination). Moreover, PPDs at 12 weeks after RD + H_2_O_2_ photolysis treatment were significantly smaller than those after treatment with RD + LDDS and RD alone (Group 3). Thus, the hypotheses were accepted, suggesting that H_2_O_2_ photolysis treatment can be beneficial when used as an adjunctive antimicrobial chemotherapy during non-surgical periodontal treatment.

Group 1 showed the highest reduction in PPD during the initial 4-week healing period. Subsequently, the PPDs in all groups were slightly reduced or maintained, and as such, the differences between the three groups remained almost unchanged up to 12 weeks. These findings suggest that RD + H_2_O_2_ photolysis treatment brings about PPD reduction during the early phase of healing. PPD reduction after periodontal treatment generally occurs as a result of 1) gingival recession, 2) clinical attachment gain, and 3) a combination of the two. In all groups, gingival recession might equally occur as a result of traumatic injury by mechanical instrumentation (RD) and resolution of swelling of gingiva. It was reported that SRP with subgingival irrigation using 3% H_2_O_2_ resulted in comparable gingival recession to SRP without irrigation^29^. This suggests that 3% H_2_O_2_ itself may not contribute to additional gingival recession. Thus, the establishment of a shallower PPD in Group 1 than that in Groups 2 and 3 might be the consequence of additional clinical attachment gain. H_2_O_2_ photolysis treatment most probably exerted maximum bactericidal effect at the base of periodontal pockets because laser light and H_2_O_2_ are released from the end of the scaler tip placed around the base during treatment. The base of the pocket is the site where re-attachment of periodontal soft tissue will start after treatment. Thus, we speculate that H_2_O_2_ photolysis treatment might create a more suitable environment for acquiring clinical attachment gain, leaving less bacteria at the base of periodontal pockets, than RD + LDDS and RD alone. Regarding BoP, there was no significant difference between Group 1 and the other groups, except for the difference between Groups 1 and 2 at the 4-week examination (P = 0.037). At the 12-week examination, BoP in Group 1 (37%) was comparable to that of Groups 2 (38%) and 3 (44%). An approximate 60% reduction in BoP 12 weeks after treatment is in accordance with results reported in the literature wherein non-surgical treatment for moderate to severe periodontitis was evaluated^7,8^.

Microbiological analysis revealed that all treatment modalities decreased the total bacterial and *P. gingivalis* counts in the periodontal pockets. Since RD is a mechanical means of removing bacteria from the root surface, it stands to reason that RD alone should lead to a substantial reduction in bacteria. Accordingly, there were no significant differences in total bacterial counts between Groups 1 and 3. In contrast, *P. gingivalis* counts in Group 1 were significantly lower than those in Group 3, and were comparable to those in Group 2. These findings demonstrate that H_2_O_2_ photolysis treatment is effective at reducing periodontal pathogens, as was LDDS, though the reduction of total bacterial counts by the RP-14 device was minimal. Since hydroxyl radicals generated by H_2_O_2_ photolysis do not diffuse over long distances^30^, the bactericidal effect via lethal oxidative damage, such as DNA oxidation and lipid peroxidation^31^, is exerted only when and where H_2_O_2_ is irradiated by laser light. As mentioned above, in the RP-14 system, H_2_O_2_ and laser light are released from the end of the scaler tip, which was placed at the base of the periodontal pocket during treatment. Hence, *P. gingivalis* inhabiting the base of the pocket would be effectively reduced in Group 1. Comparing Groups 1 and 2, the former exhibited a larger reduction in PPD than the latter, even though the reduction of *P. gingivalis* was comparable. During LDDS, antibiotics incorporated into the gel were repeatedly delivered to the periodontal pocket, which may affect the healing of periodontal tissue, thereby interfering with pocket reduction. Unlike H_2_O_2_ photolysis treatment, the bactericidal effects of 3% H_2_O_2_ alone and laser irradiation at 405 nm alone are limited, as demonstrated by pre-clinical studies^17,21,32^. Thus, bacteria inhabiting the upper portion of the periodontal pocket would not be effectively reduced. This might result in slightly higher total bacterial counts as well as significantly higher BoP at the 4-week examination in Group 1 than Group 2. The bacteria left at the upper portion of the periodontal pockets in Group 1 might prolong the inflammation of the upper portion of the gingiva, maintaining a relatively high BoP during the initial healing process. However, since patients were enrolled in a meticulous oral hygiene program, the influence of the bacteria at the upper portion of the periodontal pockets might be diminished by the supragingival plaque control in a time dependent manner. As a consequence, BoP in Group 1 became comparable to that in Groups 2 and 3 at the 12-week examination. Concerning the microbiological analysis, it should be noted that the present study dealt with only *P. gingivalis* and total bacteria. Recent advances in sequencing technologies make it possible to comprehensively analyse microbiota. Thus, to better understand how H_2_O_2_ photolysis treatment affects periodontal bacterial flora, a further study on microbiota using next-generation sequencing technology should be conducted.

RD was performed with the same amplitude and frequency of the ultrasonic scaler tips in all groups. However, since hollow- and solid-type scaler tips were used in Group 1 and the other groups, respectively, there was a difference in kinetic energy between the groups. Kinetic energy is expressed by the equation *E* = *mv* ^2^ */*2, where *E* is kinetic energy (J), *m* is the mass of the object (kg), and *v* is speed (m/s). Since the scaler tips vibrated at the same speed regardless of the type (since they had the same amplitude and frequency), the energy was proportional to the weight of the end portion of the scaler tip. The weights of the end portions of the scaler tips, calculated based on the blueprints and the gravity of the material, were 39.6 mg for the hollow-type and 77.8 mg for the solid-type. Thus, the kinetic energy of the hollow-type scaler tip was nearly half that of the solid-type. Indeed, when the acrylic resin plate was subjected to ultrasonic scaling using the hollow- and solid-type scaler tips, the weight loss caused by the former was almost half that caused by the latter. Therefore, the RD performed in Group 1 may have been less aggressive than that performed in the other groups. Nonetheless, Group 1 exhibited the lowest PPDs. This finding suggests that less-aggressive mechanical debridement would be sufficient when performed in conjunction with H_2_O_2_ photolysis treatment.

We confirmed that the safety of periodontal treatment using the RP-14 system was generally acceptable. Although two adverse events were observed (sinus membrane perforation and stomatitis of the tongue) that may have been possibly correlated to treatment using the RP-14, they were not likely related to H_2_O_2_ photolysis treatment. Perforation of the sinus membrane was more likely caused by RD than H_2_O_2_ photolysis. Since it has been demonstrated that the hydroxyl radicals generated by H_2_O_2_ photolysis do not damage the oral mucosa^26,27^, it is reasonable to assume that the perforation was caused by the mechanical intervention. However, since an influx of H_2_O_2_ into the sinus may be harmful, it is important to diagnose possible sinus membrane perforations during preoperative radiographic examination when maxillary molars require treatment. Even though the patient who experienced perforation during this study recovered without demonstrating symptoms of sinusitis, if a perforation is suspected preoperatively, treatment using the RP-14 system should be avoided. Regarding the development of stomatitis at the edge of the tongue, while it occurred close to the test tooth, it was not adjacent to the site that was directly treated with H_2_O_2_ photolysis. Although the possibility of it being correlated to the treatment using the RP-14 system cannot be denied completely, the likelihood of H_2_O_2_ photolysis treatment inducing stomatitis seems limited.

In conclusion, the clinical efficacy and safety of non-surgical periodontal therapy with H_2_O_2_ photolysis-based antimicrobial chemotherapy adjunctively performed with RD were demonstrated. Of the treatments tested, RD + H_2_O_2_ photolysis treatment achieved the shallowest periodontal pockets. Since shallow pockets facilitate maintenance of the periodontal condition after active treatment^33^, the effect of PPD reduction after RD + H_2_O_2_ photolysis treatment could be beneficial. However, as the follow-up period was only 12 weeks, a longer follow-up study should be conducted to verify the results obtained in this study. In addition, since approximately 40% of the test sites subjected to RD + H_2_O_2_ photolysis treatment exhibited inflammation (i.e., BoP positive sites), re-treatment and/or subsequent surgical treatment is still required for these cases.

## Methods

### Study design

**Figure 2:**
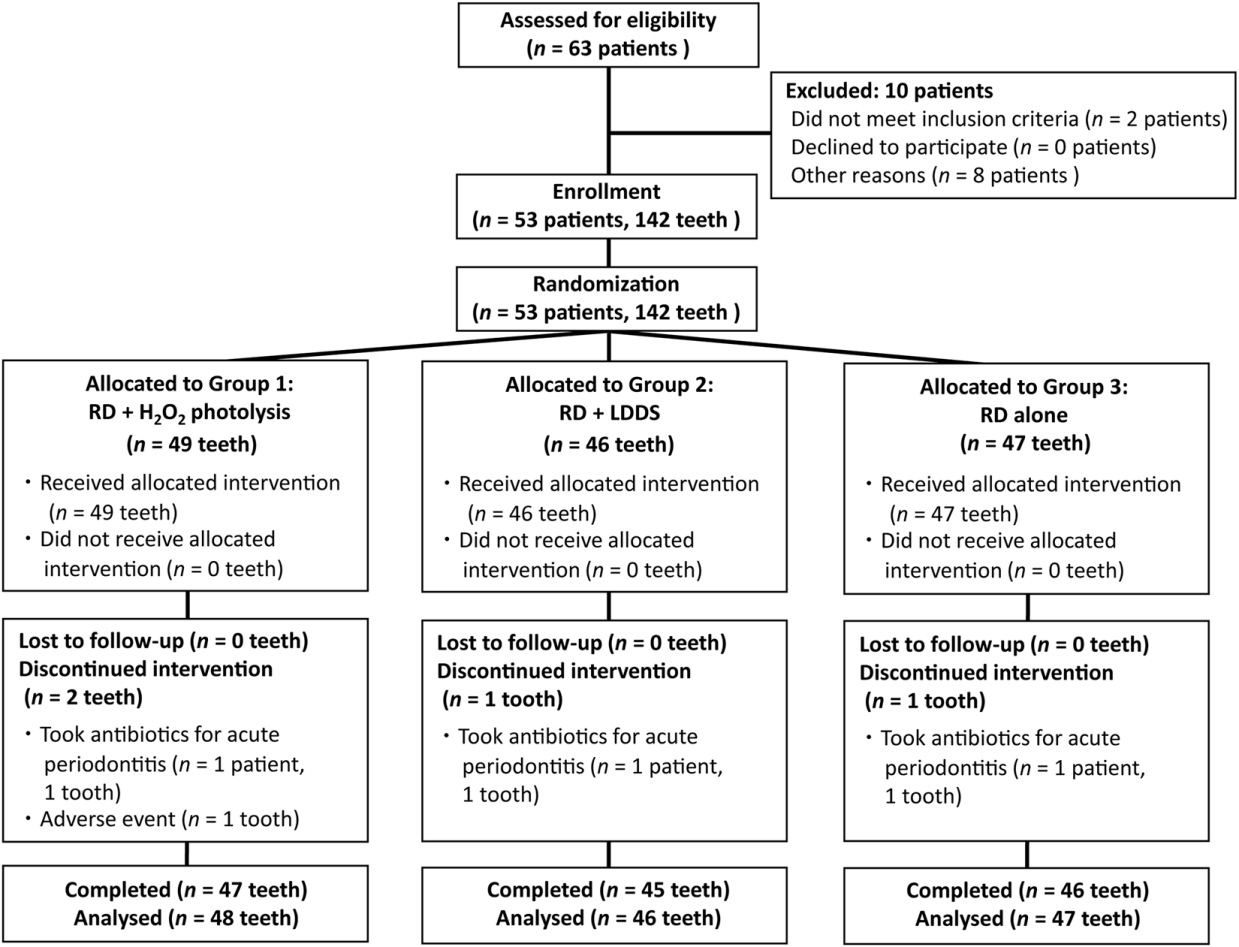
The CONSORT flow chart for this study.

This study was designed as a randomized controlled, single-blind, multi-centre (one university hospital and one private dental clinic) trial with a split-mouth design to compare the effects of non-surgical periodontal therapy with RD + H_2_O_2_ photolysis to those of RD + LDDS treatment (where RD was followed by antimicrobial treatment with LDDS) and RD alone. The research protocol was approved by the Tohoku University Hospital Institutional Review Board (IRB; reference No. 153003) and the IRB of the Kouseikai Sone Clinic. All subjects were informed about the study, given a detailed description of the procedure, and signed a written consent form. This study was performed in accordance with the Consolidated Standards of Reporting Trials (CONSORT) statement and the Good Clinical Practice (GCP) guidelines. Additionally, the study complied with the Declaration of Helsinki, as amended in Fortaleza, Brazil, in 2013. The trial was registered at the University Hospital Medical Information Network Center-Clinical Trials Registry (clinical trial registration number: UMIN000016791) on April 15, 2015. An independent data and safety monitoring board reviewed the data throughout the trial. The CONSORT flow chart of the clinical trial is presented in Fig. 2.

### Patient selection

Patients with moderate to severe periodontitis were recruited from the dental clinic at Tohoku University Hospital and a private dental clinic (Sweden Dental Sendai, Sendai, Japan). In total, 63 patients consented to be assessed for eligibility. A screening examination, including full-mouth probing and radiographic evaluation, was performed. The inclusion criteria were as follows: (i) age between 35 and 70 years, (ii) presence of at least 18 remaining teeth, (iii) diagnosis of moderate to severe chronic periodontitis^34^, (iv) presence of at least one tooth with a PPD between 6 and 8 mm, and v) BoP in two or more quadrants. The exclusion criteria were as follows: (i) smokers, (ii) having received antibiotic therapy and/or subgingival periodontal therapy in the previous 12 weeks, (iii) uncontrolled diabetes, (iv) presence of acute symptoms, (v) taking medication affecting the periodontal condition (e.g., prednisolone, phenytoin, nifedipine, or cyclosporine A), or (vi) pregnancy or breastfeeding.

### Baseline examination and randomized allocation

Baseline examination was performed one week prior to subgingival treatment, and the following variables were recorded by one examiner per facility who was masked to the randomized allocation throughout the study period: PPD, BoP, and PlI^35^; that is, the same person performed all of the baseline examinations at each facility. Probing was performed using a manual pressure-sensitive periodontal probe (Gram Probe #2, YDM, Tokyo, Japan) with a force of approximately 0.2 N at six sites/tooth. Based on the baseline examination findings, two or three test teeth (one in each quadrant) that exhibited 6 mm ≤ PPD ≤ 8 mm, BoP (+), and PlI ≤ 1 were selected from each patient. As a result, 142 test teeth were included in this study. Of the buccal, lingual, mesial, and distal surfaces of each test tooth, the surface presenting with the deepest PPD was regarded as the test site. The third molar, distal surface of the second molar, and any sites with furcation involvement were excluded. Vertical bone loss was evaluated on the radiographic images obtained at the screening examination, and a difference of ≥2 mm between the alveolar crest and the bottom of the bone defect was regarded as vertical bone loss. The test sites were randomly allocated to one of three treatment groups: Group 1, RD + H_2_O_2_ photolysis treatment; Group 2, RD + LDDS treatment; and Group 3, RD alone. Thus, the test sites in a patient were treated with different treatment modalities to compare their effects within the same individual (i.e., split-mouth design). The randomized allocation was performed by employing a minimization method using an interactive web response system, stratifying for molars or non-molars, sites with or without vertical bone loss, and the university clinic or the private clinic.

### Treatment protocol

The patients were enrolled in an oral hygiene program prior to baseline examination. They were given oral hygiene instruction, during two separate visits, for self-performed plaque control with tooth-brushing and interdental cleaning using an interdental brush and/or dental floss. At each visit, professional supragingival cleaning was also performed. The baseline examination was performed one week after the second hygiene visit.

One week after the baseline examination, non-surgical subgingival treatment was performed under local anaesthesia by dentists who were not designated as examiners. One quadrant was treated per visit, and the treatments for all quadrants were completed within 2 weeks. The test teeth were treated with one of the three non-surgical periodontal therapies (RD + H_2_O_2_ photolysis, RD + LDDS, or RD alone) using the newly developed device (RP-14, AZ. Co. Ltd, Sendai, Japan), while the remaining teeth were treated using a conventional ultrasonic scaler.

**Figure 3:**
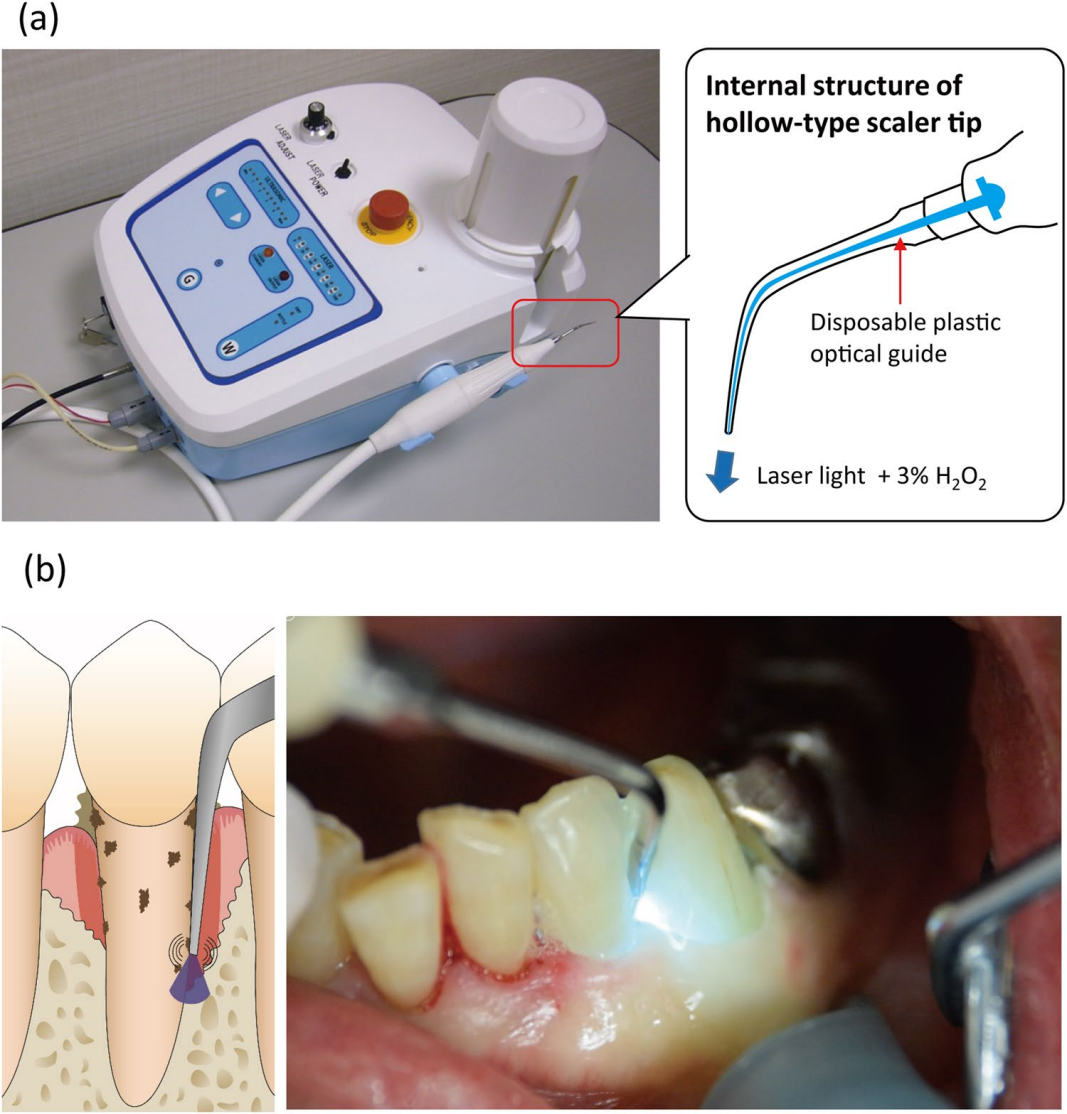
Photographic images and illustrations of the RP-14 device (**a**) used in the present clinical trial for periodontal treatment (**b**). The RP-14 is equipped with an ultrasonic scaler and a laser unit that emits light at a wavelength of 405 nm. The hollow-type, steel scaler tip and disposable plastic optical guide were used for treatment in Group 1 (root debridement + H_2_O_2_ photolysis treatment). Laser light at 50 mW and 3% H_2_O_2_ are released from the end of the scaler tip. As a result, hydroxyl radicals are generated in the periodontal pocket during root debridement.

The RP-14 was equipped with the functions of an ultrasonic scaler and a continuous-wave laser unit that emits light at a wavelength of 405 nm, as well as a water supply system to reduce the heat generated by ultrasonic scaling. A hollow-type, steel scaler tip and a disposable plastic optical guide (designed to be set inside the scaler tip) were fabricated for the device (Fig. 3). Group 1 received ultrasonic RD using the device with a hollow-type, steel scaler tip and 3% H_2_O_2_ instead of water coolant. Thus, the laser light and H_2_O_2_ were released from the end of the scaler tip during RD, which generated hydroxyl radicals as a result of a photolysis. The power output of the laser was set at 50 mW at the scaler tip using the power meter contained in the device. Groups 2 and 3 received RD using a solid-type, steel scaler tip and water coolant. The solid-type scaler tip had the same dimensions as the hollow-type, but without the hollow structure. As is the case with a conventional scaler tip, the water coolant was released from the neck portion. In all groups, RD was performed until the operator judged sufficient, or up to 7 min. Group 2 was additionally treated with an LDDS using a minocycline chloride gel (Periocline, Sunstar Inc., Takatsuki, Japan), whereas Group 3 did not receive any antimicrobial treatment. After Group 2 received RD, the antibiotic gel was injected into the periodontal pockets using a specific applicator, and gel application was repeated once per week, for three consecutive weeks (four applications in total). The patients were followed for up to 12 weeks after treatment. Throughout the study period, oral hygiene instruction and professional supragingival cleaning were performed repeatedly at every visit based on the oral hygiene status of each patient.

During laboratory testing, the amplitudes of vibration of the hollow- and solid-type scaler tips driven by the RP-14, as well as an ordinary scaler tip driven by a commercial ultrasonic scaler device (Varios750, NSK, Kanuma, Japan), were measured using a laser Doppler vibrometer (KV100-LM TYPE-D, Denshigiken, Yokohama, Japan) in accordance with the standards provided by the Japanese Industrial Standards Committee (JIS T 5750: 2009 “Dentistry – Dental handpieces – Ultrasonic instruments and tips for multi-purpose treatment”). Since the amplitude of the ordinary scaler tip used in periodontal treatment mode was 25 µm, those of the hollow- and solid-type scaler tips were set to be equivalent. The frequency of both types of scaler tips was 33 kHz, which was also set based on that of the commercial ultrasonic scaler. Furthermore, the vibration intensities of the hollow- and solid-type scaler tips driven by the RP-14 were evaluated by measuring the weight loss of an acrylic resin plate subjected to ultrasonic scaling with a load of 3.5 N for 10 min. Tests were performed using five independent assays.

### Microbiological analysis

Microbiological sampling was performed at the test sites immediately before treatment (regarded as baseline for microbiological analysis), and at 1 and 4 weeks after treatment by the blinded examiners. The sampling area was isolated and dried, and the supragingival plaque was removed. Two sterile paper points (#45, Spident, Incheon, Korea) were inserted into the test site and held in place for 30 s, then transferred into a sterile tube and sent to a contract laboratory (BML, Tokyo, Japan). Quantification of the total bacterial and *P. gingivalis* (as a representative periodontal pathogen) counts was performed by a modified Invader Plus assay applying a two-step polymerase chain reaction^36,37^. Briefly, DNA was extracted using a commercial kit (MagNA Pure LC Total Nucleic Acid Isolation Kit; Roche, Basel, Switzerland). A primer for *P. gingivalis* was designed based on genomic DNA encoding 16 S ribosomal RNA (Forward: GCGCTCAACGTTCAGCCT, Reverse: CACGAATTCCGCCTGCC). Similarly, a primary probe and an invader oligo for *P. gingivalis* were designed using Invader technology creator (Hologic, Madison, WI, USA) (Primary probe: CGCGCCGAGGGGCAGTTTCAACGGC, Invader oligo: GCCGCCGCTGAACTCAAGCCCT). In addition, a pair of universal primers (Forward: GGATTCGCTAGTAATCG, Reverse: TACCTTGTTACGACTT) and universal probe (Primary probe: CGCGCCGAGGCCGGGAACGTATTCACC, Invader oligo: TGACGGGCGGTGTGTACAAGGCA) were used to calculate the total number of bacteria. Target DNA was amplified using a thermocycler (ABI PRISM 7900, Applied Biosystems, Foster City, CA, USA), and fluorescence was detected according to a protocol provided by the manufacturer of the kit (Cleavase XI Invader core reagent kit, Hologic).

### Follow-up examination

Clinical assessments of PPD, BoP, and PlI were performed by the blinded examiners 4, 8, and 12 weeks after treatment. Only the test teeth were assessed at the 4- and 8-week examinations, whereas a full-mouth examination was performed 12 weeks after treatment. Any adverse events observed during the follow-up period were recorded. The severity of the recorded adverse events was evaluated according to the NCI CTCAE v4.0.

The primary outcome was PPD recorded 12 weeks after treatment, and the secondary outcomes were PPD at 4 and 8 weeks, BoP at 4, 8, and 12 weeks, and quantitative determination of total bacteria and *P. gingivalis* 1 and 4 weeks after treatment.

### Sample size calculation

Sample size calculation was performed to determine the number of test sites required to demonstrate the non-inferiority of RD + H_2_O_2_ photolysis treatment compared to RD + LDDS treatment with respect to the primary outcome. Based on previous studies, the difference in mean PPD between the two groups was estimated to be 0.7 mm, with a standard deviation of 1.0 mm. Providing a power of 80%, one-sided significance level of 2.5%, and non-inferiority margin of 0.3 mm, the required sample size was calculated to be 40 sites for each group. To compensate for loss to follow-up, we planned to include 46 test sites/group (138 in total) from 55 patients. When ≥46 sites/group were registered at baseline, recruitment ceased even if the number of patients was < 55.

### Statistical analysis

Statistical analyses of the data obtained in the clinical trial were performed using SAS version 9.4 (SAS Institute, Cary, NC). The non-inferiority of RD + H_2_O_2_ photolysis treatment (Group 1) on the primary outcome (PPD at the 12-week examination) in comparison with RD + LDDS treatment (Group 2), and the superiority of RD + H_2_O_2_ photolysis treatment in comparison with RD alone (Group 3) were statistically tested using the full analysis set of data (i.e., intention-to-treat analysis). If non-inferiority was verified, the superiority was additionally assessed. A general linear model was used to infer the difference in the mean PPD value at the 12-week examination between the groups. The measurement of PPD was modelled by a linear function of treatment group, and we assumed the compound symmetry covariance structure to account for the intra-subject correlations. When the lower limit of the 95% CI of the difference between the groups was greater than -0.3 mm (non-inferiority margin), Group 1 was considered not inferior to Group 2. Similarly, when the lower limits of the 95% CIs of the difference between the groups were greater than 0 mm, Group 1 was considered superior to Groups 2 or 3.

For the secondary endpoints, the statistically significant differences (P < 0.05) between the groups were also assessed using general linear models for the measurement of PPD and total bacterial and *P. gingivalis* counts, and generalized linear models for the response rate of BoP. A general linear model with time, treatment group, and the interaction term of them as explanatory variables was used. Considering the intra-subject and the inter-time correlations, the covariance structure constructed by taking the Kronecker product of an unstructured matrix with an additional compound symmetry matrix was used. Analyses of copies of bacteria were performed with logarithmically converted values. Furthermore, we used a generalized linear model with time, treatment group and the interaction term of them as explanatory variables for the response rate of BoP. The link function was “identity”, and the unstructured covariance structure was assumed to account for the inter-time correlations.

Regarding the data obtained during laboratory testing, the statistical significance (P < 0.05) of the intensity of ultrasonic vibration generated by the hollow- and solid-type scaler tips was assessed with the Student *t*-test using the JMP Pro 11.0.0 software (SAS Institute).

### Data Availability

The datasets generated and/or analysed during the current study are available from the corresponding author on reasonable request.
